# Evaluation of safety and efficacy of allogeneic adipose tissue-derived mesenchymal stem cells in pediatric bronchiolitis obliterans syndrome (BoS) after allogeneic hematopoietic stem cell transplantation (allo-HSCT)

**DOI:** 10.1186/s13287-023-03498-y

**Published:** 2023-09-19

**Authors:** Rashin Mohseni, Pouya Mahdavi Sharif, Maryam Behfar, Mohammad Reza Modaresi, Rohola Shirzadi, Mahta Mardani, Leila Jafari, Fahimeh Jafari, Zeynab Nikfetrat, Amir Ali Hamidieh

**Affiliations:** 1 Pediatric Cell and Gene Therapy Research Center, Gene, Cell and Tissue Research Institute, Children’s Medical Center Hospital, Tehran University of Medical Sciences, 63 Qarib St., Keshavarz Blvd., Tehran, 14155-6559, 1419733161 Iran; 2 Pediatric Respiratory and Sleep Medicine Research Center, Children’s Medical Center, Tehran University of Medical Sciences, Tehran, Iran; 3 Pediatric Pulmonary Disease and Sleep Medicine Research Center, Pediatric Center of Excellence, Children’s Medical Center, Tehran University of Medical Sciences, Tehran, Iran

**Keywords:** Bronchiolitis obliterans, Stem cell, Mesenchymal stem cells, Hematopoietic stem cell transplantation, Graft-versus-host disease, Transplantation, Lung, Clean room

## Abstract

**Background:**

Allo-HSCT is a definite approach for the management of a wide variety of lethal and debilitating malignant and non-malignant disorders. However, its two main complications, acute and chronic graft-versus-host disease (GVHD), exert significant morbidities and mortalities. BoS, as a manifestation of chronic lung GVHD, is a gruesome complication of allo-HSCT, and for those with steroid-refractory disease, no approved second-line therapies exist. Mesenchymal stem cells (MSCs) exert anti-inflammatory and growth-promoting effects, and their administration against a wide range of inflammatory and neurologic disorders, as well as GVHD, has been associated with promising outcomes. However, literature on the safety and effectiveness of MSC therapy for BoS and pediatric cGVHD is scarce.

**Methods:**

We designed a single-arm trial to administer adipose tissue (AT)-derived MSCs to pediatric patients with refractory BoS after allo-HSCT. AT-MSCs from obese, otherwise healthy donors were cultured in an ISO class 1 clean room and injected into the antecubital vein of eligible patients with a dose of 1 × 10^6^/kg. The primary endpoints included a complete or partial response to therapy [in terms of increased forced expiratory volume in one second (FEV1) values and steroid dose reduction] and its safety profile.

**Results:**

Four eligible patients with a median age of 6.5 years were enrolled in the study. Steroid-induced osteoporosis and myopathy were present in three cases. A partial response was evident in three cases after a single injection of AT-MSCs. The treatment was safe and tolerable, and no treatment-related adverse events were noted. Two patients developed manageable COVID-19 infections one and 4 months after AT-MSC injection. After a median follow-up duration of 19 months, all cases are still alive and have had no indications for lung transplantation.

**Conclusions:**

AT-MSCs could be safely administered to our pediatric cases with BoS post-allo-HSCT. Considering their advanced stage of disease, their sub-optimal functional capacity due to steroid-induced complications, and COVID-19 infection post-treatment, we believe that AT-MSC therapy can have possible efficacy in the management of pediatric BoS. The conduction of further studies with larger sample sizes and more frequent injections is prudent for further optimization of AT-MSC therapy against BoS.

*Trial registration* Iranian Registry of Clinical Trials (IRCT), IRCT20201202049568N2. Registered 22 February 2021, https://en.irct.ir/trial/53143.

**Supplementary Information:**

The online version contains supplementary material available at 10.1186/s13287-023-03498-y.

## Background

Allogeneic hematopoietic stem cell transplantation (allo-HSCT) is a curative strategy against various debilitating malignant and non-malignant disorders [1–3], but it is complicated by the development of acute and chronic graft-versus-host disease (aGVHD and cGVHD, respectively) [4, 5]. With improved survival rates of aGVHD, more than 50% of such cases and roughly 30–70% of all allo-HSCT recipients develop cGVHD [6]. Corresponding statistics for pediatric patients are less consistent and, depending on the source and method of performing allo-HSCT, between 6 and 65% of all and 18 and 27% of aGVHD cases are being diagnosed with cGVHD [7, 8]. Despite having lower mortality rates [9], cGVHD poses a significant morbidity and economic burden and is associated with considerable compromise in the quality of life of affected individuals and an increased incidence of infections, respiratory failure, and intensive care unit admissions [9–11]. It becomes particularly cumbersome for pediatric cases, as their significantly prolonged survival will be associated with such complications [5]. Lung involvement is among the more difficult-to-treat manifestations of cGVHD and is associated with more profound morbidity and mortality [9].

Bronchiolitis obliterans (BO) is the most common and deleterious form of the late-onset noninfectious pulmonary complication (LONIPC) of allo-HSCT, which is reported to afflict about 3.7–11% and 10–14% of all allo-HSCT and cGVHD cases, respectively [12–14]. BO is also associated with increased mortality in allo-HSCT and lung transplant patients [12–14]. While its exact pathological underpinnings are not deciphered yet, aberrations in innate and adaptive immune system activation and excessive tissue fibrosis (as the hallmarks of cGVHD and BO) have been documented [6, 15, 16]. The definite diagnosis of BO lies in the pathological assessment of lung biopsy samples. However, owing to the invasiveness nature of lung biopsy, the National Institutes of Health (NIH) has proposed criteria for the clinical diagnosis of bronchiolitis obliterans syndrome (BoS, which denotes the lack of pathological evaluations), which is widely accepted by authors [17, 18]. Current general approaches for the management of BoS comprise a short trial of pulsed corticosteroids with a rapid taper and fluticasone–azithromycin–montelukast [FAM] combination, and, in refractory cases, they include extracorporeal photopheresis (ECP), other immunosuppressives, and lung transplantation (as the last resort) [19–27]. However, these approaches are not studied in pediatric cases and are not robustly effective against progressive BoS [20, 28].

Mesenchymal stem/stromal cells (MSCs) are multipotent cells that can be obtained from various tissues (including bone marrow [BM], adipose tissue [AT], umbilical cord, and placenta) [29–31], and apart from regenerative capacities, they harbor immunomodulatory and growth-promoting characteristics [32–35]. Experimental [36, 37] and clinical [38–41] studies have demonstrated their effectiveness in the treatment of GVHD, and their low and no expression of human leukocyte antigens (HLA) class I and class II, respectively, make them ideal targets for allogeneic transplantation [42]. Compared to other sources, AT-derived MSCs (AT-MSCs) are easy to obtain [43], have less genomic instability and senescence than BM-derived MSCs [44], harbor higher proliferation capacities [37, 45], and are superior in inducing anti-inflammatory effects and surviving after transplantation [37, 45, 46]. Accordingly, it has recently been shown that these features can empower AT-MSCs to demonstrate better protection against aGVHD (compared to BM- and umbilical cord-derived MSCs) [37].

As there are no established second-line therapies for steroid-refractory BoS, we designed a phase I single-arm trial to evaluate the safety and efficacy of AT-MSCs against post-allo-HSCT refractory BOS in pediatrics. Given the beneficial impacts of AT-MSCs and the pathological underpinnings of BoS, we hypothesized that AT-MSCs can effectively resolve or control the progression of BoS after allo-HSCT.

## Methods

### Study design and patient selection

We designed an open-label, uncontrolled, non-randomized trial to evaluate the safety and efficacy of allogeneic AT-MSCs for the management of BoS in pediatric (< 18 years) patients who underwent allo-HSCT at the stem cell transplantation unit of Children Medical Center Hospital and had diagnosed with BoS between October 2020 and April 2022. The inclusion and exclusion criteria for the enrollment of eligible cases are presented in Table 1. The diagnosis of BoS was made based on the 2015 modified NIH criteria, which was initially released in 2005 [17, 18]. These criteria have been recognized as the gold standard tool for the diagnosis of BoS [23] and are widely accepted by relevant authorities [47].

**Table 1:** Inclusion and exclusion criteria for patient enrollment

*Inclusion criteria*
Age < 18 years
Receiving allo-HSCT within a year before enrollment
Signs and symptoms of BoS (dyspnea on exertion, dry cough, wheezing, pneumothorax, pneumomediastinum, and subcutaneous emphysema) [17] Evidence of air trapping or small airway involvement in HRCT
Definite diagnosis of end-stage, steroid-refractory BoS, according to the modified NIH criteria [17]*: First, the FEV1/FVC or SVC must be less than 0.7 or the 5th percentile of predicted (with appropriate adjustments for pediatric or elderly individuals) Second, FEV1 must be less than 75% of the predicted value, with at least a 10% decline over less than 2 years Third, respiratory tract infections must be ruled out Fourth, there must be evidence of either air trapping in expiratory CT scans, or air trapping in pulmonary function tests (RV more than 120% of the predicted values or RV/TLC increased more than 90% confidence interval), or small airway thickening or bronchiectasis in HRCT
*Exclusion criteria*
BoS caused by any etiology other than allo-HSCT
Evidence of relapsed or progressive underlying malignant disorder
Evidence of viral, bacterial, or fungal pneumonia
HLA-haploidentical or T-cell-depleted transplantation
Known history of allergy or adverse drug reactions

*Allo-HSCT* Allogeneic hematopoietic stem-cell transplantation, *BoS* Bronchiolitis obliterans syndrome, *cGVHD* chronic graft-versus-host disease, *CT* computed tomography, *FEV1* forced expiratory volume in 1 s, *FVC* forced vital capacity, *HLA* human leukocyte antigen, *HRCT* high-resolution computed tomography, *NIH* national institutes of health, *RV* residual volume, *SVC* slow vital capacity, *TLC* total lung capacity

*All four criteria must be present to make a diagnosis of BoS. However, in cases with established cGVHD, the presence of the first three findings suffices BoS diagnosis

Two expert pediatrics pulmonologists and two expert pediatrics stem cell transplantation specialists were responsible for the assessment of cases and confirmation of their eligibility. Due to the experimental nature of the study and ethical considerations, patients were allowed to continue receiving other prescribed medications for their allo-HSCT (prednisolone, ECP, and other immunosuppressive agents). We took written informed consent from AT donors and each patient’s parents after the detailed clarification of the experimental nature of the trial and the anonymity of enrolled individuals. This study is undertaken in accordance with the ethical principles and guidelines of the Declaration of Helsinki and is approved by the ethical committee of the Tehran University of Medical Sciences (ethics registration code, IR.TUMS.MEDICINE.REC.1399.406). The findings of this study are reported according to the Consolidated Standards of Reporting Trials (CONSORT) recommendations.

### MSC preparation

AT was obtained from obese, otherwise healthy, HLA-unrelated, third-party donors who underwent liposuction at university-affiliated centers (their baseline characteristics are demonstrated in Additional file 1: Table S1). The acquisition, expansion, and characterization of MSCs were conducted following the accredited protocols [48] and under good manufacturing practice (GMP) conditions. A detailed explanation of performed procedures is depicted in the Additional file 1: Detailed steps of adipose tissue-derived mesenchymal stem/stromal cells preparation.

### Procedure

Patients received a single injection of AT-MSCs with a dose of 1 × 10^6^/kg in their antecubital (median cubital) veins. In addition, if the weight of patients exceeded 35 kg, the total dose was divided into two separate injections at 2-days intervals. Intravenous (IV) injections were conducted with cardiopulmonary monitoring, and patients remained in close monitoring for a minimum of 12 h post-injection. Patients and their parents were instructed to visit the pediatrics HSCT and pulmonology clinics for timely follow-ups (F/Us) and go to the pediatric emergency centers upon the occurrence of adverse events (fever, seizure, anaphylaxis, skin rashes, palpitation, chest pain, dyspnea, etc.).

### Outcome measure and endpoints

The endpoints of this study were the safety and efficacy of the application of AT-MSCs to pediatric patients with allo-HSCT-induced BoS. Spirometry was performed before and at 1-, 3-, and 6-months intervals after the injection of AT-MSCs and was interpreted by a pediatrics pulmonologist. High-resolution computed tomography (HRCT) of the chest was taken before and at 1- and 6-months intervals after the injection of AT-MSCs, for a minimum. Changes in the %forced expiratory volume in one second (%FEV1), FEV1/forced vital capacity (FVC), and HRCT features were recorded for each patient. According to the NIH consensus [49], an increase in the %FEV1 of 10% predicted or more was considered a partial response (PR), and an increase of %FEV1 to more than 80% was considered a complete response (CR). A diagnosis of progressive disease was made upon a 10% or more decrease in the absolute to predicted value of %FEV1 [49]. In addition, and according to the descriptions of previous trials [50], we defined a reduction in the steroid dose by at least 50% (without disease progression) as a PR. The grading of adverse events was performed according to the Common Terminology Criteria for Adverse Events (CTCAE), version 6.0.

## Results

After evaluating 12 pediatric cases with a diagnosis of BoS post-allo-HSCT, four patients (one female, Table 2) met the eligibility criteria, consented to enroll in the trial, and participated in subsequent follow-up visits. Three cases were diagnosed with acute lymphocytic leukemia (ALL), and one case had thalassemia major. In addition, two had been diagnosed with manageable aGVHD. The characteristics of their allo-HSCT are depicted in Table 2.

**Table 2:** Characteristics of the allo-HSCT procedure

Recipient features			Donor features		HSCT characteristics								
Pt. no	Age	Sex	Age	Sex	Donor type	ABO status	SC source	Conditioning regimen	Graft volume (cc)	MNC dose/kg (× 10^8^)	CD3^+^ dose/kg (× 10^8^)	CD34^+^ dose/kg (× 10^6^)	GVHD prophylaxis
1	16	M	15	M	MSD	Matched	PB	BuCy	500	7.5	3.86	2.8	CyA/MTX
2	6	M	35	M	MSD	Mismatched	PB	BuCy + ATG	106	8.0	4.21	4.4	CyA/MTX
3	7	F	13	M	MSD	Mismatched	PB	BuCy	150	8.0	2.17	5.4	CyA/MTX
4	4.5	M	21	F	MSD	Matched	PB	BuCy	99	8.0	1.96	9.7	CyA/MTX

*ATG* Anti-thymocyte globulin, *BuCy* busulfan plus cyclophosphamide, *CD* cluster of differentiation, *CyA/MTX* cyclosporine and methotrexate, *F* female, *GVHD* graft-versus-host disease, *Kg* kilograms, *M* male, *MNC* mononuclear cell, *MSD* matched sibling donor, *PB* peripheral blood, *SC* stem cell

**Fig. 1:**
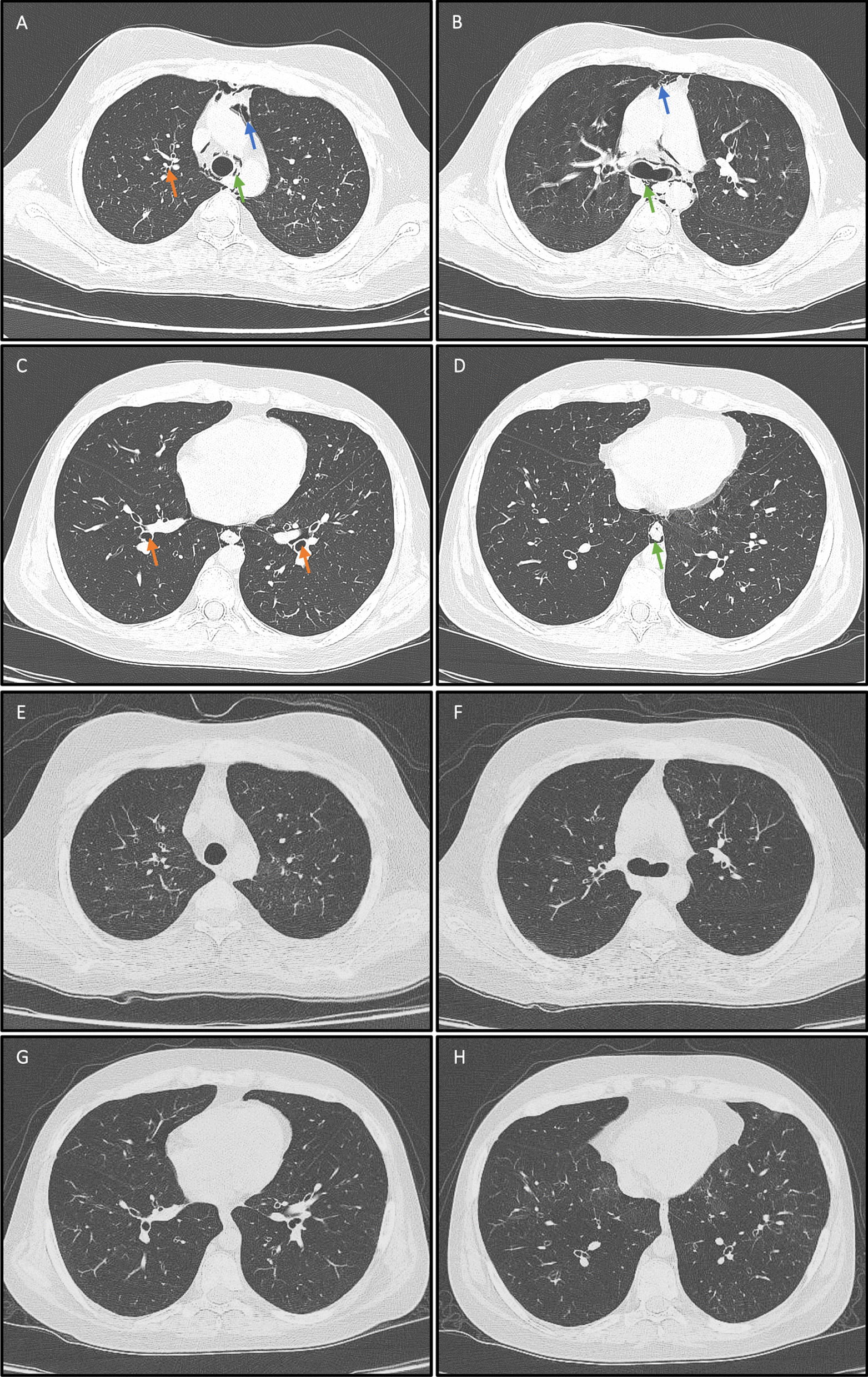
Chest CT scan images of the first patient before (panels **A**–**D**) and 12 months after (panels **E**–**H**) treatment with AT-MSCs. Resolution of bilateral lung hyperaeration, pneumomediastinum (*green arrows*), pneumopericardium (*blue arrows*), and to a lesser extent, bronchiectasis (*orange arrows*) is evident

The first patient was a 16-year-old boy who was diagnosed with ALL. He received a fully matched allo-HSCT from his brother and, about 6 months later, presented with signs and symptoms in favor of BoS (Table 3). Two months after this diagnosis, and after observing no improvements in response to conventional regimens, he received AT-MSCs with a dose of 1 × 10^6^/kg. One month after the MSC injection, his spirometry parameters were stable (Table 4), and prednisolone and mycophenolate mofetil (MMF) were tapered to 15 mg/d and 250 mg/d, respectively. At the 6-months F/U, he was on prednisolone (10 mg/d), while cyclosporine and MMF were discontinued (Table 3). He also showed a promising response to AT-MSCs in chest CT image sections (Fig. 1 and Table 5).

**Table 3:** Detailed prescribed treatments before the receiving of AT-MSCs and at the last F/U visit

Pt. no	Hx of aGVHD	BoS therapies before AT-MSC	allo-HSCT-AT-MSC interval (mo)	Treatment(s) at the last F/U	F/U duration (mo)	CMV infection post-therapy	Status
1	No	PDN (20 mg/d), cyclosporine (75 mg/d), MMF (500 mg/d), and 14 ECP sessions	8	PDN (10 mg, two times per week), ruxolitinib (5 mg/d), and 8 ECP sessions	13	No	Alive
2	Grade III skin and GI	PDN (15 mg/d) and tacrolimus (0.5 mg/d)	18	Ruxolitinib (2 mg/d), tacrolimus (0.5 mg, two times per week), and 15 ECP sessions	20	No	Alive
3	Grade II skin	PDN (25 mg/d), tacrolimus (1 mg/d), and MMF (600 mg/d)	12	PDN (10 mg/d), MMF (250 mg/d), tacrolimus (0.5 mg/d), and 19 ECP sessions	19	No	Alive
4	No	PDN (15 mg/d), sirolimus (1 mg/d), and MMF (125 mg/d)	9	PDN (12.5 mg/d), ruxolitinib (5 mg/d), and 18 ECP sessions	19	No	Alive

*aGVHD* acute graft-versus-host disease, *allo-HSCT* allogeneic hematopoietic stem-cell transplantation, *AT-MSC* adipose tissue-derived mesenchymal stem/stromal cells, *BoS* bronchiolitis obliterans syndrome, *CMV* cytomegalovirus, *d* day, *ECP* extracorporeal photopheresis, *F/U* follow-up, *GI* gastrointestinal, *Hx* history, *mg* milligrams, *MMF* mycophenolate mofetil, *mo* months, *PDN* prednisolone

**Table 4:** Details of the PFT results of included cases

Pt. no	Parameter	PFT (% to predicted, months)							
		− 2	0	1	3	6	8	12	21
1*	FEV1	N/A	29	35	35	33	N/A	N/A	N/A
	FEV1/FVC		45	42	42	41			
	SpO_2_ (%)		95	96	97	95			
2*	FEV1	N/A	28	28	33	32	38	32	23
	FEV1/FVC		45	41	45	42	50	55	51
	SpO_2_ (%)		92	93	96	96	96	96	94
3	FEV1	35	N/A	45	43	N/A	N/A	N/A	N/A
	FEV1/FVC	47		45	44				
	SpO_2_ (%)	96†		96	96				
4*	FEV1	42	N/A	38	34	32	N/A	N/A	N/A
	FEV1/FVC	74		75	54	51			
	SpO_2_ (%)	98†		95	94	95			

*FEV1* forced expiratory volume in 1 s, *FVC* forced vital capacity, *N/A* not available, *no* number, *PFT* pulmonary function test, *SpO*_*2*_, oxygen saturation

*Documented evidence of corticosteroid-induced osteoporosis

†With oxygen supplementation

**Table 5:** HRCT findings of included cases before and after the administration of mesenchymal stem cells

Pt. no	HRCT findings (months)
1	− 2: PM and thin fibrotic bands in LUL 0: PM, PP, and SE (suggestive of cGVHD) + 6: Bilateral lung hyperaeration, bronchiectasis, and PT + 12: Mild hyperaeration, bronchiectasis, and PT
2	− 5: PT and bilateral GGOs at the middle and inferior lung portions + 5: Mild, central bronchiectasis
3	0: Moderate and diffuse bronchiectasis and lung hyperaeration + 4: Mild, bilateral bronchiectasis and lung hyperaeration, mild PM, PP, and SE, and a thin-walled cavity at the upper pole of RLL; 2 and 4 weeks later, progression of PM, PP, and SE was noted (possibly due to underlying COVID-19) + 18: Same, without obvious progression or resolution of abnormalities
4	− 3: Bilateral PT and hyperaeration, with minimal MA (in favor of bronchitis) 0: Diffuse bilateral MA with UL predilection, in favor of cGVHD + 1: MA and patchy GGOs in both lungs, diffuse PT, and mild bronchiectasis, probably due to COVID-19; subsequent images taken 2 weeks later showed mild bronchiectatic changes in both LLs + 4: Diffuse hyperaeration, MA, bronchiectasis, bronchial thickening, mucus plugs, and GGOs in both ULs + 6: LL dominant bronchiectatic changes and MA* in both lungs, mucus plaque formation, and an 11 × 11 mm pneumatocele in RUL with thin adjacent fibrotic bands (all in favor of cGVHD) + 12: Bronchiectatic changes, bronchial wall thickening, scattered centriacinar nodules, and MA* in both lungs + 20: Cylindrical bronchiectasis in both LLs with some bronchoceles and MA in both lungs

*cGVHD* chronic graft-versus-host disease, *GGO* ground glass opacity, *HRCT* high-resolution computed tomography, *LL* lower lobe, *LUL* left upper lobe, *MA* mosaic attenuation, *PM* pneumomediastinum, *PP* pneumopericardium, *PT* peribronchial thickening, *RLL* right lower lobe, *RUL* right upper lobe, *SE* subcutaneous emphysema, *UL* upper lobe

^*^In favor of small airway disease

The second patient, a six-year-old boy with ALL, received one locus mismatched (9/10) allo-HSCT from his father. Approximately 70 days after transplantation, he presented with grade II skin and gastrointestinal aGVHD. The treatment of aGVHD was successful; however, chronic limited skin GVHD persisted. Six months after transplantation, he displayed insidious symptoms suspicious for BoS, and 5 months later, a definite diagnosis of BoS was made by pediatrics pulmonologists (Table 3). Observing minor clinical benefits, he received AT-MSCs 7 months after the diagnosis of BoS. Despite the lack of response to AT-MSCs in early scheduled F/Us, his %FEV1 showed a 10%-increase 8 months after MSC therapy (Table 4). His prednisone was tapered to 5 mg, and with the initiation of ruxolitinib (1 mg/d) and ECP, steroid therapy was discontinued. In the last F/U (20 months), despite a decrease in %FEV1 (23%, Table 4), his only complaint was dyspnea on exertion, and the blood oxygen saturation was 99%.

**Fig. 2:**
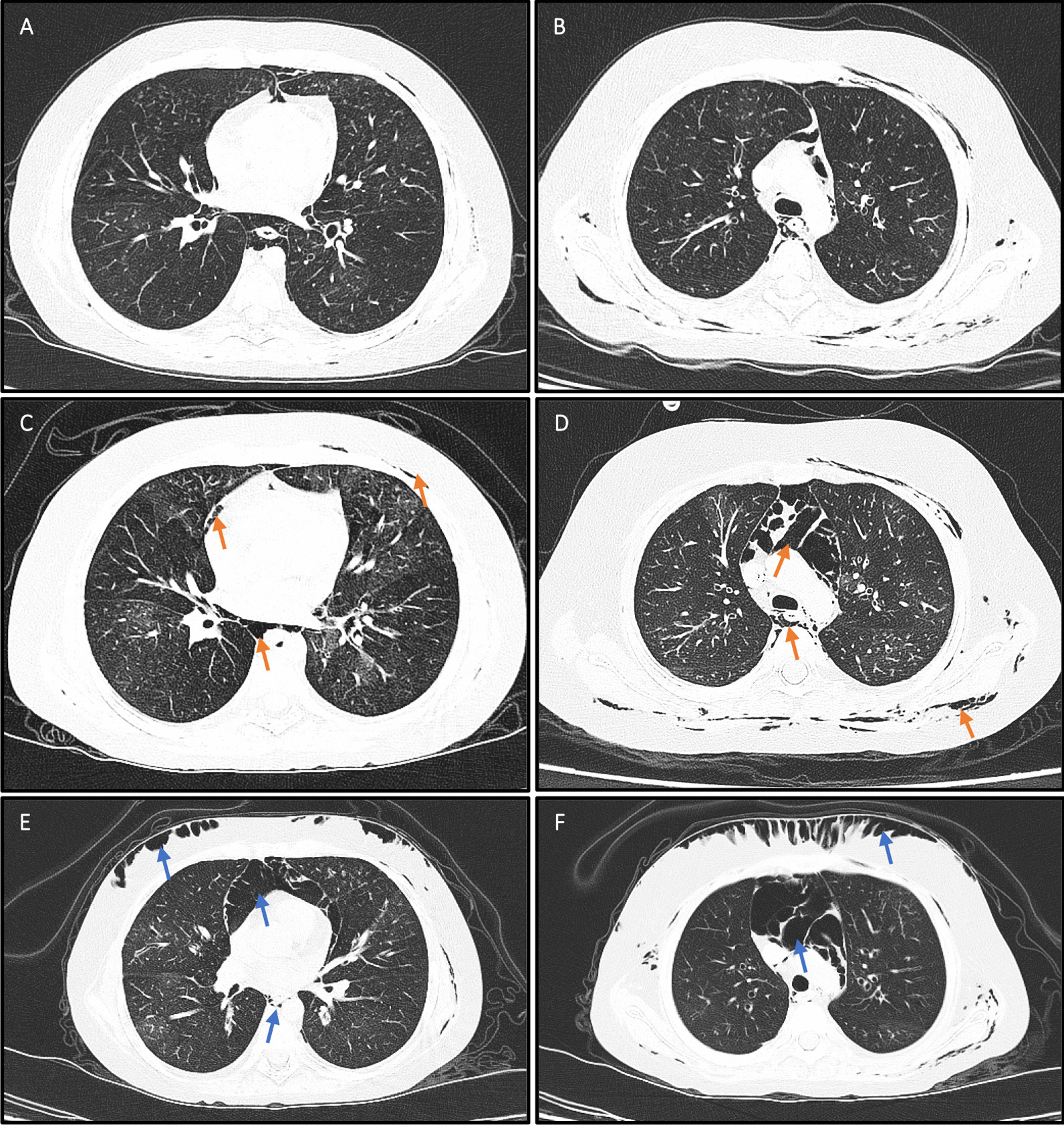
Chest CT images of the third case, who developed COVID-19 infection four months after AT-MSC therapy (panels **A** and **B**). Two weeks later, pneumomediastinum, pneumopericardium, and subcutaneous emphysema (*orange arrows*) progressed (panels **C** and **D**); however, these abnormalities did not show a significant progression (*blue arrows*) in images obtained 12 months later (panels **E** and **F**)

The third case is a seven-year-old girl with ALL who received a fully matched allo-HSCT from his brother. She developed manageable grade II skin aGVHD. About 6 months after her allo-HSCT, she started to present trivial yet progressive respiratory distress and a decrease in blood oxygen saturation. Treatment with conventional approaches was initiated, and upon their lack of efficacy, AT-MSCs were applied 6 months after the diagnosis of BoS (Table 3). In the 1-month F/U after the injection of AT-MSCs, her FEV1 showed a 10% increase (Table 4), her cyanosis resolved, and her respiratory distress improved partially. Four months after AT-MSC therapy, she developed Coronavirus disease 2019 (COVID-19) infection, and her chest-CT images showed the development and expansion of pneumomediastinum, pneumopericardium, and subcutaneous emphysema (Table 5). However, these complications did not progress in subsequent evaluations (Fig. 2), and on the last F/U (19 months), she had received 19 sessions of ECP and had significant tapering in her immunosuppressive doses (Table 3).

The last patient was a known case of thalassemia major who underwent a fully matched allo-HSCT (from his sister) at the age of 4.5 years. About 7 months after allo-HSCT, he exhibited the presentations of BoS. After the failure of conventional therapies in controlling BoS, 2 months after the BoS diagnosis, he received AT-MSCs (Table 3). One month after the administration of AT-MSCs, he developed signs and symptoms of an acute respiratory infection, and upon hospitalization, COVID-19 infection was confirmed. This infection was manageable, and despite the deterioration in chest CT findings (Table 5) at the 2-months F/U after AT-MSC therapy, his spirometry parameters remained stable, and he was weaned from supplementary oxygen therapy. However, his symptoms began to deteriorate, and his spirometry values worsened at the 6-months F/U. As a result, despite an initial taper in prednisolone (7.5 mg/d) and MMF (250 mg, two times per week), he re-maintained on prednisolone (20 mg/d) and ruxolitinib (5 mg/day) 12 months after AT-MSC therapy, and tacrolimus (1 mg/d), and ECP were added to his therapeutic regimen. At the last F/U, tacrolimus was discontinued, and prednisolone was tapered to 12.5 mg/d (Table 3). It should be noted that this patient was diagnosed with steroid-induced myopathy, which might inversely affect the %FEV1.

In summary, the F/U duration for the first case was 13 months, and the other three have been followed for at least 19 months post-AT-MSC therapy. In our evaluations, all treated patients had clinical evidence of improvement in respiratory functions. As such, two cases (#2 and #3) were weaned from supplemental oxygen, and all demonstrated improvements in their daily activities. Moreover, excluding one case (#4), all other patients had at least a 50% decrease in their steroid doses. However, due to the safety concerns and allowance of concomitant administration of other treatments (namely ruxolitinib and ECP), we could not robustly determine whether this steroid-sparing benefit was a direct impact of AT-MSC therapy.

Overall, the treatment was safe and tolerable in all cases. None of them experienced infusion toxicity or adverse drug reactions. Likewise, no treatment-related adverse events occurred after AT-MSC injections. Notably, COVID-19 infections developed 6 and 9 months after the official announcement of COVID-19 spread in the country, when repetitive surges were occurring, and no approved vaccine for pediatrics was available in the country. As a result, we believed that no treatment-related infectious complications developed. Lastly, no relapse of underlying ALL has been observed yet.

## Discussion

In this study, we described the preliminary evidence on the safety and efficacy of the first-time AT-MSC therapy for the management of BoS in pediatric patients who had received allo-HSCT. We found that despite sub-optimal objective responses in terms of an increase in %FEV1, eligible cases exhibit possible clinical improvements following this therapy.

As briefly mentioned, MSCs harbor anti-inflammatory and growth-promoting features that are exerted through various distinct paths [51–53]. Earlier studies exhibited that MSCs are effective in preventing acute lung injury, inflammation, and fibrosis following exposure to endotoxin [54] and bleomycin [55, 56] and can alleviate collagen deposition in lung tissue [53, 54, 56–58].

Most reports on the efficacy of MSC therapies against BoS are limited to lung transplantation in adults. In a phase I single-arm study on ten cases with chronic lung allograft dysfunction and BoS, the IV administration of BM-MSCs (8 × 10^6^/kg, divided into four infusion sessions) was able to meaningfully (but not significantly) diminish the decline in FEV1 values [59]. Another phase Ia study on nine cases with moderate, treatment-refractory BoS after lung transplantation did not observe any benefits from BM-MSC therapy (1 to 4 × 10^6^/kg) in the 1-month F/Us [60]. Nevertheless, in this study’s subsequent phase Ib trial on 13 lung transplant patients with moderate-to-severe BoS, allogenic BM-MSCs (0.5 or 1 × 10^6^/kg) were effective in ceasing the significant reductions in FEV1 and FVC at the 12-months F/U, implicating the long-term efficacy of single-dose MSC therapy in preserving PFT parameters [61].

While the application of MSC therapy in the management of aGVHD is well-discussed in the literature [38–40, 62, 63], evidence of its efficacy for cGVHD is scarce. By and all, the outcomes have been promising, even in those with severe steroid-refractory disease [64–67]. However, some studies have documented conflicting observations on the response of cGVHD (including lung involvement) to autologous BM- [68] and allogeneic umbilical cord-derived MSCs [69]. In fact, in the Stenger et al. [68] study, none of the two adult cases displayed a response in their lung involvement. On the other hand, Shen et al. found that of three cases with lung cGVHD, one obtained a CR, one obtained a PR, and the third case displayed a stable disease [69] at the 3-months F/U.

Concerning allo-HSCT cases with BoS, a phase I/II study [50] enrolled 81 patients (aged between 18 and 59 years) with known BoS to investigate the efficacy of treatment with allogeneic BM-MSCs. Patients were non-randomly allowed to receive either azithromycin and prednisone or their combination, along with MSCs. The initial dose of MSCs was 4 × 10^6^/kg (divided into four infusion sessions), and another 4 × 10^6^/kg was allowed for those who had responded to the initial cycle. This study found that at the 3-months post-enrollment evaluations, 71% in the MSC group versus 44% in the non-MSC groups had a response (defined as an increase in FEV1 or > 50% steroid dose reduction) [50]. However, the differences lost their significance in those with severe BoS. In addition, while the differences in FEV1 stabilization/increase and the estimated 3 years overall survival were not significant between the two groups, MSC therapy was superior in facilitating a steroid dose reduction by at least 50% and mitigating the reduction in FEV1 from baseline [50]. Last but not least, there are inconsistent observations on the considerable enhancements in PFT parameters of allo-HSCT cases with BoS following umbilical cord MSC therapy [70].

Our study reported the efficacy and safety of first-time AT-MSCs administration for the management of pediatric BoS. Regarding the efficacy of this therapy, the objective outcomes were somehow heterogeneous. Two patients had an early (1-month) PR to AT-MSC therapy, while the PR of the third case was observable 8 months later. In addition, the first case exhibited considerable improvements at his 12-months F/U HRCT images.

Of note, despite clinical stability, the second case exhibited deteriorations in PFT parameters at the 12-months F/U. Besides, the last case did not respond to the therapy and exhibited a gradual decrease in %FEV1. However, he had severe steroid-induced osteoporosis (with a *Z*-score of − 4.1) and documented myopathy with evident gait dysfunction. Nevertheless, as mentioned earlier, all cases showed evidence of considerable improvements in respiratory functions and symptoms. Of note, discrepancies between the clinical picture and spirometry findings might stem from the fact that spirometry cannot be reliably performed in preschool children [71, 72], which was the scenario for three of our included cases. Likewise, it is suggested that FEV1 is not a robust indicator of the respiratory function of children [49]. Added to this, at least three of our cases had evidence of steroid-induced osteoporosis and myopathy, which can profoundly attenuate respiratory muscle functions and, consequently, spirometry performance [73].

This study faces several limitations. First, due to the low incidence of BoS, the refractory disease of the included cases, and the experimental and cell-based nature of the study, we did not add a second or control arm to the study. Second, in concordance with the protocols of previous trials and with consideration of safety measures in pediatric patients, we injected AT-MSCs for one time and at the dose of 1 × 10^6^/kg, which theoretically can result in suboptimal activities of MSCs. Third, the continuation of other treatments was allowed during enrollment. Although none of the included cases were responsive to the conventional treatments for cGVHD, the interaction of these therapies with MSCs cannot be reliably ruled out. Fourth, due to safety concerns and a lack of enough data on MSC therapy in pediatrics, our study was limited to only four cases, which hinders its solid generalizability and the drawing of robust conclusions. Initially, there were twelve identified cases with BoS after allo-HSCT; however, evidence of latent tuberculous infection in the chest HRCT images of three cases and lack of consent to participate in five cases made them ineligible for enrollment in the trial. In addition, one case (#3) refused to perform all scheduled PFTs. Lastly, two patients (#3 and #4) developed COVID-19 infection after the administration of AT-MSCs, and although their infection was manageable, its negative impacts on pulmonary functions might have played a role in the lack of an acceptable clinical response to AT-MSC injection.

## Conclusions

In conclusion, this study found that intravenous administration of AT-MSCs is a safe and tolerable approach, with promising subjective and acceptable objective efficacy in controlling BoS and preventing its deterioration following allo-HSCT in pediatrics. In fact, after a median F/U duration of 19 months after the administration of AT-MSCs, all of them have remained alive and are still in no need of lung transplantation. The four enrolled cases in our trial had a definite diagnosis of BoS according to the 2015 modified NIH criteria, and accordingly, all pediatric patients who suffer from BoS and meet these criteria might benefit from allogeneic AT-MSC therapy. Compared to other sources of MSCs, AT-MSCs are easier to retrieve, culture, and preserve and are superior in exerting immunomodulatory and growth-promoting influences. Subsequent studies with larger sample sizes and more frequent injections are required to robustly delineate the efficacy of MSC therapies in different grades of BoS.

### Supplementary Information

**Additional file 1:** Detailed steps of adipose tissue-derived mesenchymal stem/stromal cells preparation.

## Data Availability

All data generated or analyzed during this study are included in this published article and its Additional file 1 on the detailed steps of adipose tissue-derived mesenchymal stem/stromal cells preparation and the characteristics of AT-MSC donors.

## Abbreviations

allo-HSCT: Allogeneic hematopoietic stem cell transplantation
aGVHD: Acute graft-versus-host disease
cGVHD: Chronic graft-versus-host disease
JAK: Janus kinase
BO: Bronchiolitis obliterans
LONIPC: Late-onset noninfectious pulmonary complication
NIH: National Institutes of Health
BoS: Bronchiolitis obliterans syndrome
FAM: Fluticasone–azithromycin–montelukast
ECP: Extracorporeal photopheresis
MSC: Mesenchymal stem/stromal cell
BM: Bone marrow
AT: Adipose tissue
HLA: Human leukocyte antigens
AT-MSC: Adipose tissue-derived mesenchymal stem cell
HRCT: High-resolution computed tomography
FEV1: Forced expiratory volume in 1 second
FVC: Forced vital capacity
RV: Residual volume
SVC: Slow vital capacity
TLC: Total lung capacity
CONSORT: Consolidated Standards of Reporting Trials
GMP: Good manufacturing practice
IV: Intravenous
Kg: Kilograms
F/U: Follow-up
PR: Partial response
CR: Complete response
CTCAE: Common Terminology Criteria for Adverse Events
ALL: Acute lymphocytic leukemia
ATG: Anti-thymocyte globulin
BuCy: Busulfan plus cyclophosphamide
CD: Cluster of differentiation
CyA/MTX: Cyclosporine and methotrexate
MNC: Mononuclear cell
MSD: Matched sibling donor
PB: Peripheral blood
MMF: Mycophenolate mofetil
mg: Milligrams
COVID-19: Coronavirus disease 2019
CMV: Cytomegalovirus
PDN: Prednisolone
GI: Gastrointestinal
Hx: History
N/A: Not available
PFT: Pulmonary function test
SpO2: Oxygen saturation
GGO: Ground glass opacity
LL: Lower lobe
LUL: Left upper lobe
MA: Mosaic attenuation
PM: Pneumomediastinum
PP: Pneumopericardium
PT: Peribronchial thickening
RLL: Right lower quadrant
RUL: Right upper quadrant
SE: Subcutaneous emphysema
UL: Upper lobe
IL: Interleukin
TNF-α: Tumor necrosis factor-alpha
IFN-γ: Interferon-gamma
MIP: Macrophage inflammatory protein
NK cell: Natural killer cell
Treg: Regulatory T-cell
Breg: Regulatory B-cell
