# Supplementary material for: Evaluation of safety and efficacy of allogeneic adipose tissue-derived mesenchymal stem cells in pediatric bronchiolitis obliterans syndrome (BoS) after allogeneic hematopoietic stem cell transplantation (allo-HSCT)
Source: Stem Cell Res Ther. 2023 Sep 19;14:256. doi: 10.1186/s13287-023-03498-y (PMC10510238; doi:10.1186/s13287-023-03498-y)
Supplement: Supplementary file 1 — Additional file 1: Detailed steps of adipose tissue-derived mesenchymal stem/stromal cells preparation. [file 13287_2023_3498_MOESM1_ESM.docx]

**Evaluation of Safety and Efficacy of Allogeneic Adipose Tissue-Derived Mesenchymal Stem Cells in Pediatric Bronchiolitis Obliterans Syndrome (BoS) after Allogeneic Hematopoietic Stem Cell Transplantation (allo-HSCT)**

**Supplementary Material**

**Detailed steps of adipose tissue-derived mesenchymal stem/stromal cells preparation**

Obtained subcutaneous abdominal adipose rinsed in Hanks' Balanced Salt Solution (HBSS) and phosphate-buffered saline (PBS) five times and was incubated with 0.05% Collagenase I (Sigma-Aldrich, USA) for 30 minutes at 37°C. Suspensions were centrifugated at a speed of 500 X g (Hettich Rotina 380R) for five minutes, and the resulting precipitates (stromal vascular fraction) were filtered through the 70 μm cell strainers. After the addition of PBS, the solution was centrifuged again at a speed of 500 X g for ten minutes, and precipitates were then incubated (Eppendorf^®^ New Brunswick™ Galaxy^®^ 170 S CO_2_ Incubator, Germany) with low-glucose Dulbecco’s modified Eagle’s Medium (L-DMEM, Gibco, USA), 10% fetal bovine serum (FBS, Gibco, USA), and 100 U/ml of penicillin-streptomycin-amphotericin B (Gibco, USA) for 24 hours under a humidified atmosphere at 37°C, 5% CO_2_ conditions. After 24 hours, the medium was replaced to remove nonadherent cells, and this was repeated every 24 hours until the cells reached 90% confluence. Thereafter, cells were washed with PBS three times, and 500 μl of Trypsin/EDTA solution (0.5%, Biowest, France) was added for cell detachment. Pipetting was repeated until a homogenous suspension ensued, which underwent centrifugation at a speed of 500 X g for ten minutes. Cells were then passaged to a maximum of three cell culture flasks (Corning, USA), and after four days, cultured cells were separated to undergo quality assessment for intravenous injection. The quality assessment consisted of trypan blue staining for cell viability assay, flow cytometry for the detection of MSC markers (CD73^+^CD90^+^CD105^+^ and CD34^-^CD45^-^; Cellul Baft Azma Laboratory, Tehran), inspection by invert microscope for morphological evaluation, and screening for pathogenic contaminations (mycoplasma, CMV, human immunodeficiency virus, and hepatitis B and C virus; Keyvan Virology Laboratory, Tehran).

| **Supplementary Table 1.** Characteristics of AT-MSC donors | | | | | | | |
| --- | --- | --- | --- | --- | --- | --- | --- |
| **Donor** | **Age** | **Gender** | **Body mass index** | **Fasting plasma glucose** | **Hypertension** | **Cardiovascular disease** | **Lipid profile** |
| **#1** | 35 | Female | 39 | 99 | No | No | Hypercholesterolemia |
| **#2** | 41 | Female | 36 | 94 | No | No | Hypercholesterolemia |
